# Four-section approach of fetal congenital heart disease at 11–13^+6^ weeks

**DOI:** 10.3389/fcvm.2023.1206042

**Published:** 2023-08-24

**Authors:** Wen Ling, Qiumei Wu, Shan Guo, Shangqing Li, Hong Ma, Biying Huang, Liqin Zeng, Tingting Dang, Min Liu, Xiuqing Qiu, Zongjie Weng

**Affiliations:** ^1^ Department of Medical Ultrasonics, Fujian Maternity and Child Health Care Hospital, College of Clinical Medicine for Obstetrics & Gynecology and Pediatrics, Fujian Medical University, Fuzhou, China; ^2^ Department of Pathology, Fujian Maternity and Child Health Care Hospital, College of Clinical Medicine for Obstetrics & Gynecology and Pediatrics, Fujian Medical University, Fuzhou, China; ^3^ Department of Obstetrics & Gynecology, Fujian Maternity and Child Health Care Hospital, College of Clinical Medicine for Obstetrics & Gynecology and Pediatrics, Fujian Medical University, Fuzhou, China

**Keywords:** fetal congenital heart disease, four-section approach, ultrasound, early trimester, image pattern

## Abstract

**Objective:**

The objective of the study is to explore the value of the four-section approach in detecting fetal heart defects in the first trimester (11–13^+6^ weeks), analyze the reasons for the inconsistency between the results of ultrasound examination in the first trimester and subsequent verification, and describe the most common abnormal flow patterns of four sections.

**Materials and methods:**

Between June 2019 and June 2021, a prenatal four-section approach (upper abdominal transverse section, four-chamber section, three vessel–trachea section, and bilateral subclavian artery section) with verification results in early pregnancy was analyzed.

**Results:**

In total, 9,533 fetuses were included. Finally, 176 fetuses with congenital heart disease (CHD), containing 34 types, were identified. The total detection rate of cardiac abnormalities was 1.85%. 102 cases were accurately diagnosed by ultrasonography during early pregnancy. A total of 74 fetuses who had inconsistent results between fetal cardiac ultrasound and verification in early pregnancy were reported, of which the cases of 22 fetuses were inconsistent due to disease evolution and progression and the cases of 52 fetuses were inconsistent due to missed diagnosis and misdiagnosis. The sensitivity, specificity, positive predictive value, and negative predictive value of the four-section approach were 67.05%, 99.96%, 96.58%, and 99.33%, respectively. In this study, a total of 30 abnormal ultrasonic imaging patterns in four sections were summarized.

**Conclusion:**

We confirmed that the four-section approach in early pregnancy has a good diagnostic efficacy for fetal CHD. Intrauterine evolution of the fetal heart, missed diagnosis, and misdiagnosis are the reasons for the inconsistency between the results of early pregnancy ultrasound and subsequent verification. This study also presents the abnormal imaging patterns of four scan sections of CHD in early pregnancy, which are instructive for the rapid identification and diagnosis of CHD in the first trimester.

## Introduction

Congenital heart disease (CHD) involves the formation of the heart and large blood vessels in the early stages of embryonic development disorders, resulting in structural and functional defects in the fetal heart and main vessels (1). CHD is the most common congenital defect (2). The incidence rate of CHD among live births in China is approximately 8.98%, and among these cases, the incidence rate of death in the neonatal period or of critical CHD requiring intervention is 1.46% (3). Studies have shown that prenatal diagnosis of CHD in early pregnancy can significantly improve the perinatal outcome (4). The exploration of CHD screening and diagnostic methods in early pregnancy to identify affected fetuses has become an area of interest that is also challenging. Many studies have shown that observation of the fetal heart in early pregnancy is feasible (5–8), and the four-chamber section (4CV section) and the three vessel–trachea section (3VT section) have been proposed to be the most important basic sections for cardiac screening in the first trimester (9–11). The 4CV section has a sensitivity of 26.0%–45.7% for CHD in early pregnancy (12–14). The 3VT section can also provide anatomical information such as the morphology, size, and route of the two large arteries, which is an effective alternative for the outflow tract section in early pregnancy. The 3VT section improves the detection rate of abnormally large blood vessels (10). Most current studies have used 4CV and 3VT sections to screen the fetal heart in early pregnancy (10, 14–17). In practice, we have found that the upper abdominal (UAb) transverse and bilateral subclavian sections are significantly feasible during early pregnancy. The upper abdominal transverse section plays an important role in determining the position of the fetal visceral atrium. Bilateral subclavian artery (BSa) sections can show the relationship between bilateral subclavian arteries and the trachea, and these two sections can be used as supplements of 4CV and 3VT sections. While the diagnostic efficacy of the combination of these four sections has not been confirmed, few data are available on the appearance of the four-section views in fetuses with specific structural cardiac defects in the first trimester. The factors affecting the accuracy of fetal cardiac ultrasonography in early pregnancy also need to be further studied.

Therefore, this study used the four-section approach (including upper abdominal transverse section, 4CV section, 3VT section, and bilateral subclavian artery section) to examine the fetal heart during early pregnancy (11–13^+6^ weeks). We compared fetal echocardiography with the verification results and evaluated the diagnostic efficacy of the four-section approach in early pregnancy. Moreover, we analyzed the reasons for the inconsistency between the results of ultrasound examination in the first trimester and subsequent verification, demonstrated the abnormal ultrasonic imaging pattern of fetal CHD in early pregnancy, and described their association with specific CHD types, which could provide more evidence for clinical decision-making.

## Material and methods

### Study population

Between June 2019 and June 2021, 22,615 pregnant women were screened for nuchal translucency (NT) examination in the Department of Ultrasound Medicine at Fujian Maternity and Child Health Care Hospital. A total of 9,533 pregnant women met the inclusion criteria. The pregnant women who at the same time met criteria (1), (4), and (2) or (3) were included in the study: (1) NT screening was performed in our hospital, and the four-section approach was completed; (2) pathological examinations such as cardiac micropathological anatomy during early pregnancy, heart topographic anatomy, and cardiovascular casting during middle pregnancy were completed; (3) fetal echocardiography was performed in our hospital during pregnancy, delivery was catered in our hospital, and neonatal congenital heart disease screening was completed; and (4) only singleton pregnancy was considered.

The average age of pregnant women was 27.5 ± 3.8 years, and the gestational age of the fetal scan was 11–13^+6^ weeks. Pregnant women and their families have been informed and agreed to the study, which was approved by the Ethics Committee of Fujian Maternity and Child Health Care Hospital (2018-017).

### Study protocol

By using GE Voluson E8 or E10 color Doppler ultrasound diagnostic instrument, the frequency of the transabdominal ultrasound probe is 4–5 MHz. The frequency of cavity probe is 6–10 MHz. The frequency of high-frequency linear array probe is 8–12 MHz. Fetal NT and early pregnancy cardiac examination mode were selected for screening.

According to the International Society of Ultrasound in Obstetrics and Gynecology (ISUOG) Fetal Ultrasound Guidelines for Early Pregnancy (18), routine transabdominal ultrasound was performed, and transvaginal ultrasound was also performed, when necessary, in combination with the fetal examination protocol of the center at 11–13^+6^ weeks of gestation.

#### Four-section approach at 11–13^+6^ weeks

**Figure 1:**
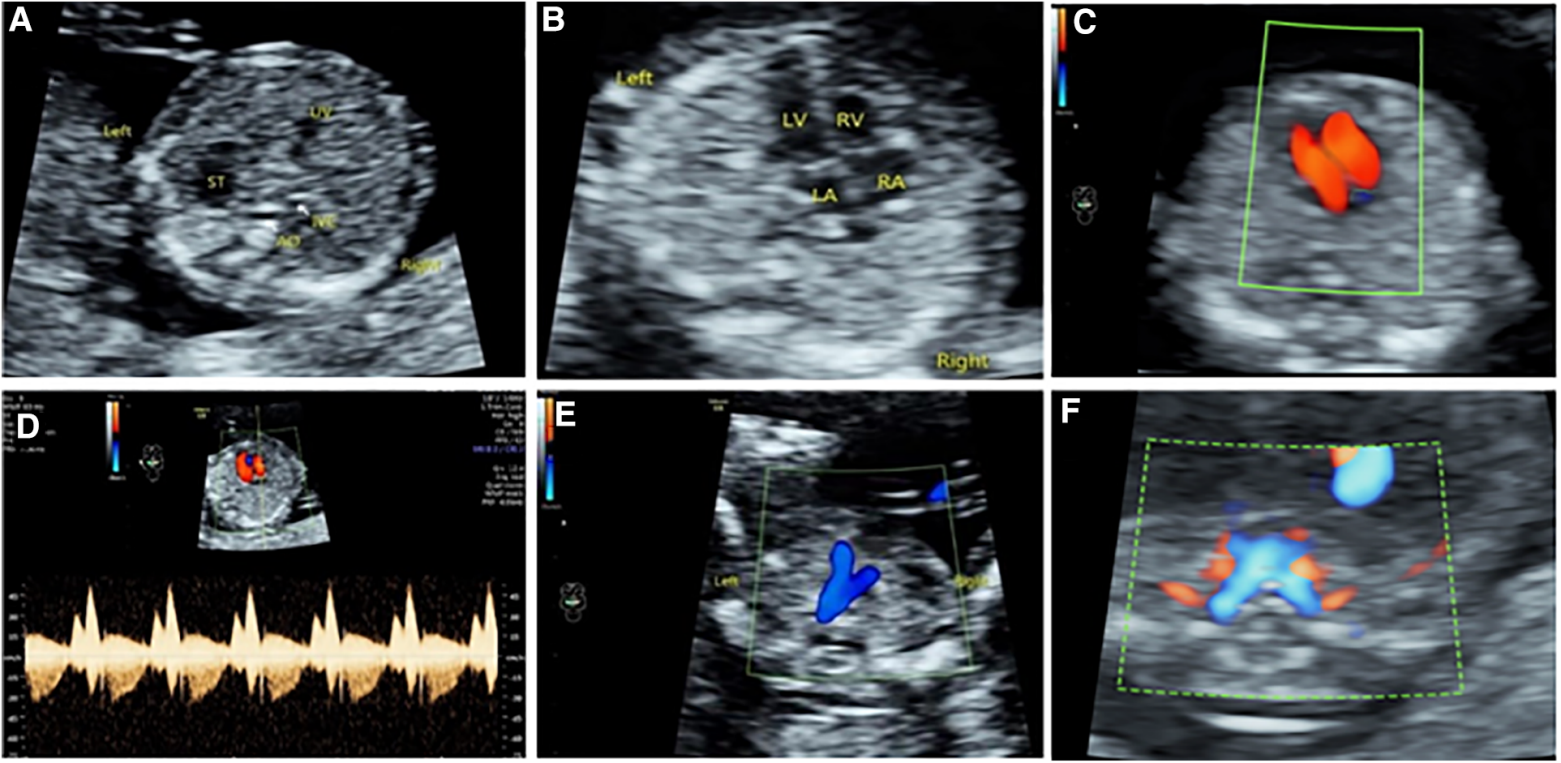
Four-section approach in early pregnancy. (**A**) Upper abdominal transverse section. (**B**–**D**) Two-dimensional, color Doppler, and tricuspid spectrum Doppler ultrasound of 4CV section. (**E**) 3VT section. (**F**) Bilateral subclavian artery section.

The acquisition includes upper abdominal transverse section, 4CV section, 3VT section, bilateral subclavian artery section, two-dimensional or superimposed color Doppler flowing imaging (CDFI), static blood flow, and arterial images (Figure 1). Upper abdominal transverse section: ultrasound scanning method—the acoustic beam was scanned through the gastric bubble and liver in the fetal upper abdomen, and the upper abdominal transverse section could be obtained. Observations: (1) the integrity of the abdominal wall, (2) the main structures of the upper abdominal transverse section (gastric vesicles, abdominal segment of umbilical vein, abdominal aorta, inferior vena cava), and their positions to judge the position of internal fetal organs. Four-chamber section: ultrasonic scan method—based on the examination of the upper abdominal transverse section, the probe was shifted to the head side to display the four-chamber incisal plane, and the ultrasonic beam was directed to the interventricular septum at approximately 45°. This not only ensured ventricular blood flow filling but also allowed the two-dimensional heart structure to be most clearly displayed. Observations: (1) the heart was located in the left chest cavity, and the apex of heart was facing to the left, (2) the heart cross structure, (3) combined with two-dimensional and color Doppler to observe whether the size of the heart cavity was symmetrical, and (4) whether there was tricuspid regurgitation. Three vessel–trachea section: ultrasonic scan method—based on the color Doppler ultrasound examination of the four-chamber section, the acoustic beam was tilted to the fetal head side to obtain the color Doppler vessel–trachea section. According to the actual situation, the incidence angle of acoustic beam was adjusted to achieve the optimization of the image: (1) the position relationship between the pulmonary artery and the aorta was determined, (2) the inner diameter of the pulmonary artery and the aorta were symmetrical, and (3) the ductus arteriosus and the aortic arch blood flow were normal. Bilateral subclavian artery section: ultrasound scanning method—based on the color Doppler ultrasound examination of the three vessel–trachea section, the acoustic beam continued to the fetal cephalic deflection, then the bilateral subclavian artery section was obtained, and the image was optimized by means of energy Doppler or adjusting the incidence angle of the acoustic wave. Observations: bilateral subclavian artery route.

#### Verification of congenital heart disease

The routine ultrasound examination of the fetal heart in the second and third trimesters adopts the nine-slice sequential segmentation analysis method of the fetal heart (19). The routine fetal heart screening in the third trimester adopts the 4CV and 3VT sections. When fetal CHD was identified, further genetic suggestions were proposed. For cases of termination of pregnancy, malformation micropathological anatomy, heart topographic anatomy or cardiovascular casting were selected according to the size of the gestational week and the type of malformation.

The pathological anatomy of the fetal heart was mainly conducted by the method of *in situ* observation after formalin fixation and *in vivo* or *in vitro* autopsy. The cast specimen was established by combined chest and abdominal cavity casting (19). In the case of labor induction during the second trimester, a single large cardiac blood vessel was cast *in vivo*. The specimens were photographed and archived, and the casting results were recorded. The cases of fetuses with CHD whose mothers continued their pregnancy routinely underwent nine-section cardiac ultrasound during the second trimester. If CHD is identified, it is necessary to recommend fetal echocardiography and provide perinatal management and prognosis counseling. A pediatrician clinically examines all births for neonatal CHD, and those screened positive for neonatal CHD infants with prenatal CHD should undergo neonatal echocardiography. When the screening result of CHD is normal after birth, the heart structure is considered normal.

#### Identification and classification of fetal CHD

Determination of fetal heart abnormalities in early pregnancy—the presence of one of the following three conditions in fetal echocardiography during early pregnancy can be considered abnormal: (1) indication of a specific CHD, (2) suspicion of specific CHD, and (3) discovery of cardiac minor lesions such as ventricular ratio imbalance, aortic ratio imbalance, abnormal cardiac position, and valvular regurgitation that cannot be determined. The abnormal statistics did not include persistent left superior vena cava, patent foramen ovale, atrial septal defect, and patent ductus arteriosus. Classification principle: fetal CHD is classified according to the main malformation. For example, coarctation of the aorta with ventricular septal defect is classified as coarctation of the aorta. When an atrioventricular septal defect and a double outlet right ventricle coexist, it is classified as atrioventricular septal defect. The diagnosis of the major malformation is correct. Then, the case diagnosis is considered correct. In addition, first-trimester diagnoses that are modified in later pregnancy or initial abnormalities evolve, and the classification of the CHD is based on the final diagnosis. According to different segments of the heart, CHD is divided into the following five categories: abnormal internal and cardiac positions, abnormal inflow tract, abnormal outflow tract, abnormal aortic arch and its branches, and other abnormalities.

### Statistical analysis

SPSS 19.0 software was used for the statistical analysis of data. For the statistical description of quantitative data, mean ± standard deviation (x ± S) is used, frequency is used for qualitative data (*n*), and percentage (%) is expressed. The receiver operating characteristic (ROC) curve was drawn to calculate the area under the curve (AUC), sensitivity, specificity, positive predictive value, and negative predictive value of each indicator, to evaluate the diagnostic effectiveness of different section approaches. The DeLong test compared multiple ROC curves.

## Results

**Figure 2:**
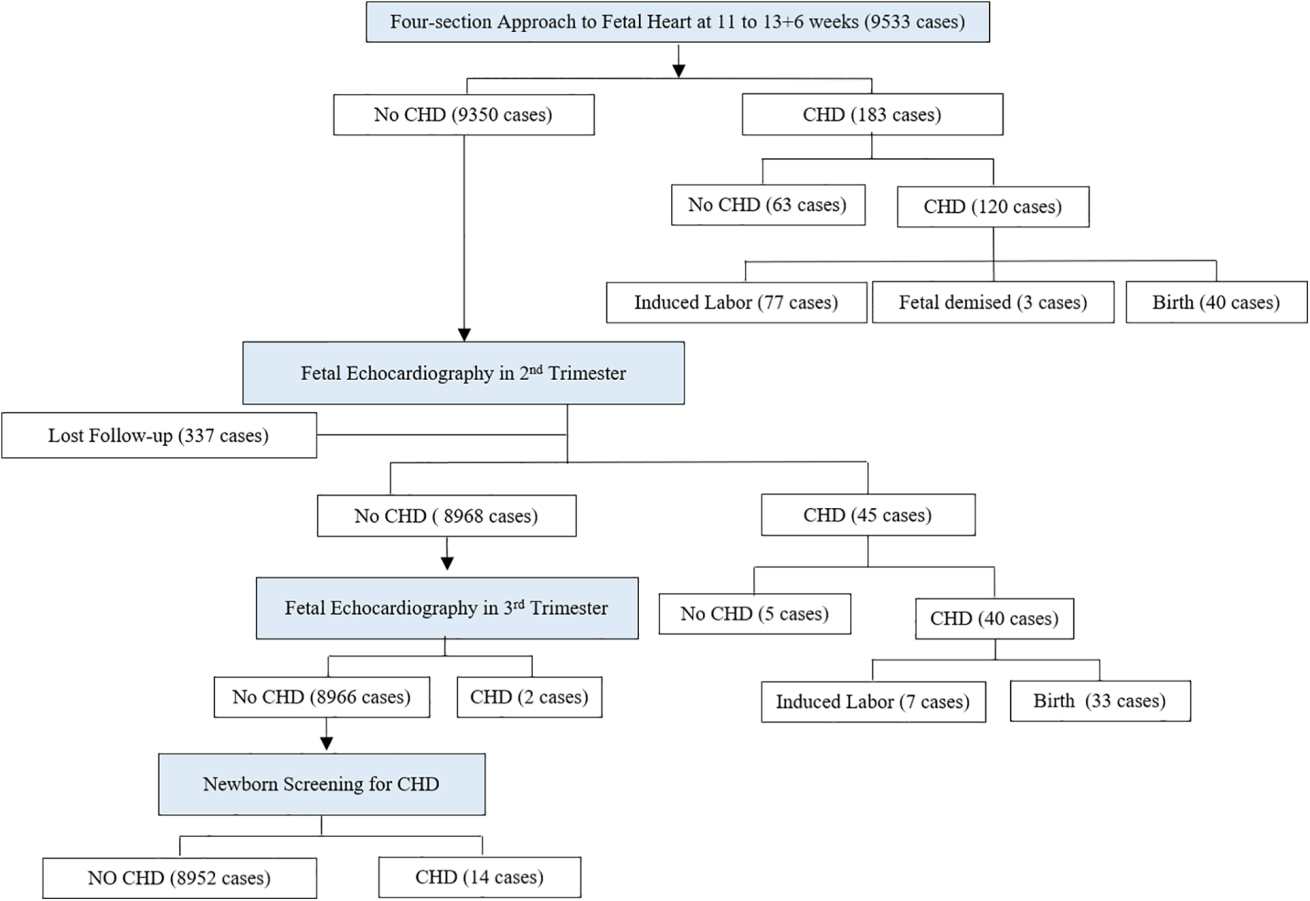
Flow chart of this cohort.

A total of 34 types of cardiac abnormalities and 176 fetuses were examined through pathological and clinical verification, and the total detection rate of cardiac abnormalities was 1.85%. In total, 183 fetuses with abnormal heart were identified by ultrasound during early pregnancy (including 61 fetuses with tricuspid regurgitation), of which 120 fetuses were subsequently confirmed to have CHD and 63 fetuses were excluded (including 59 fetuses with tricuspid regurgitation). A total of 9,350 fetuses with normal heart were identified by ultrasound in the first trimester, of which 56 fetuses with CHD were noted in a follow-up screening (40 fetuses were identified in the second trimester, two fetuses were identified in the third trimester, and 14 fetuses were identified in the neonatal period), and 9,294 fetuses were reported as normal, as presented in Figure 2.

### Diagnostic efficacy of four-section approach

**Figure 3:**
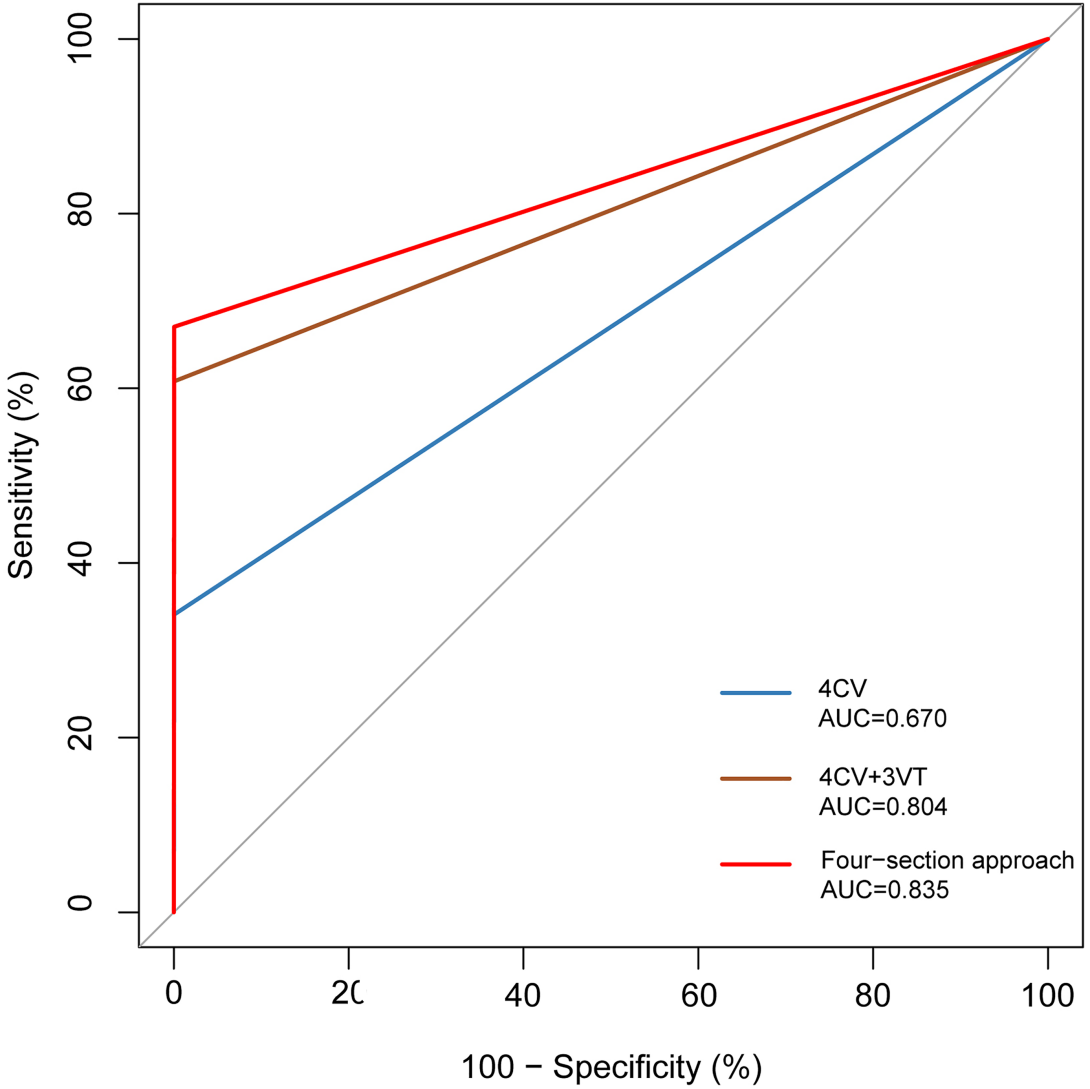
ROC curve comparison between different section approaches for diagnosis of CHD.

In the 176 fetuses with CHD in this study, 5.68% (10/176) had an abnormal upper abdominal transverse section, 34.09% (60/176) had an abnormal 4CV section of the heart, 53.41% (94/176) had abnormal 3VT section, and 19.89% (35/176) had abnormal bilateral subclavian artery section. Approximately 67.04% (118/176) of the fetuses were reported to be abnormal in at least one section, and 60.23% (106/176) were abnormal in 4CV section combined with 3VT section, presented in Table 1. The upper abdominal transverse section was most found in an abnormal fetal visceral heart position (88.88%, 8/9), abnormal 4CV section was most found in the abnormal inflow tract (78.26%, 18/23), and abnormal 3VT section was most found in the abnormal outflow tract (71.70%, 38/53). Abnormalities in bilateral subclavian artery sections were most common in the aortic arch and its branch anomalies (27/53, 50.94%). The 4CV section, 4CV section combined with 3VT section, and the four-section approach were used to evaluate heart defects in early pregnancy. The AUC values of 4CV section, 4CV section combined with 3VT section, and the four-section approach had significant statistical differences (all *p* < 0.01). AUC values, sensitivity, specificity, and positive and negative predictive values of the diagnosis of CHD are presented in Table 2 and Figure 3.

**Table 1:** Cardiac abnormality by different section approaches.

	Total	Type of complicated heart abnormality	Ab	4CV	3VT	2SA	4CV + 3VT
CHD	**176**		**10**	**60**	**94**	**35**	**106**
Abnormal position of organs and the heart	**9**		**8**	**9**	**8**	**2**	**9**
Ectopia cordis	2	/	2	2	2	2	2
Displacement of the heart	1	/		1			1
Dextrocardia	2	Situs inversus × 1, situs inversus + VSD × 1	2	2	2		2
RAI	3	Dextrocardia + AVSD + PS + TAPVC × 1; AVSD + PA + TAPVC × 1 AVSD + DORV + PS × 2	3	3	3		3
LAI	1	AVSD + PS + AVB + interruption of IVC × 1	1	1	1		1
Abdominal inflow tract	**23**	** **	**0**	**18**	**15**	**1**	**18**
AVSD	9	COA × 1; DORV + PS × 1; PS × 2		7	4		7
MS	1	/					
MA	1	VSD + DORV × 1		1	1		1
HLHS	3	/		3	3		3
TA	1	VSD + PS × 1		1	1		1
HRHS	4	RAA × 1, ARSA × 1		4	4	1	4
TVD	1	/					
Ebstein’s anomaly	3	PS × 2		2	2		2
Abnormal outflow tract	**53**	** **	**1**	**16**	**38**	**5**	**38**
TOF	16	RAA + ALSA × 3, RAA × 2, CASV × 1		6	12	3	12
TGA	6	Mesocardia + VSD × 1; VSD × 3, VSD + PS × 2		3	6		6
DORV	5	Dextrocardia + levoversion of heart + VSD + PS × 1; VSD + PS × 2;PS × 2	1	2	5		5
PA-VSD	7	RAA × 1, RAA + ALSA × 2		2	7	2	7
PA-IVS	2	VCAC × 1		1	1		1
CASV	7	Enlarged cardiothoracic ratio × 1; VSD × 1; VSD + COA × 1		2	7		7
PS	10	VSD × 2					
Abnormal aortic arch	**53**	** **	**0**	**11**	**33**	**27**	**35**
ARSA	26				13	22	13
CoA	14	VSD × 2; BAV × 1		10	8		10
RAA	8	ALSA × 6, MIRAA × 1			7	5	7
DAA	2				2		2
IAA	1	VSD × 1			1		1
AA	1	Dextroversion of the heart + ARSA × 1		1	1		1
APW	1				1		1
Other	**38**	** **	**1**	**6**	**0**	**0**	**6**
VSD	30	Situs inversus × 1	1	5			5
PAS	2	UHPA × 1		1			1
TAPVC	2						
RLINV	1						
NVM	1	VSD × 1					
CAF	1						
CR	1						
Normal heart	9357		0 (0%)	4 (0.04%)	0 (0%)	0 (0%)	4 (0.04%)

RAI, right atrial isomerism; AVSD, atrioventricular septal defects; PS, pulmonary stenosis; TAPVC, total anomalous pulmonary venous connection; PA, pulmonary atresia; DORV, double outlet right ventricle; LAI, left atrial isomerism; AVB, bicuspid aortic valve; IVC, inferior vena cava; COA, coarctation of aorta; MS, mitral stenosis; MA, mitral atresia; VSD, ventricular septal defect; HLHS, hypoplastic left heart syndrome; TVD, tricuspid valve dysplasia; TA, tricuspid atresia; RAA, right aortic arch; ARSA, aberrant right subclavian artery; TOF, tetralogy of Fallot; ALSA, aberrant left subclavian artery; CASV, congenital absence of semilunar valve; TGA, transposition of great arteries; PA-VSD, pulmonary atresia with ventricular septal defect; PA-IVS, pulmonary atresia with intact ventricular septum; DAA, double aortic arch; IAA, interrupted aortic arch; AA, aortic atresia; APW, aortopulmonary window; PAS, pulmonary artery sling; RLINV, retroaortic left innominate vein; NVM, non-compaction of ventricular myocardium; CAF, coronary artery fistula; CR, cardiac rhabdomyomas.

CHD was divided into five categories in our study, bold values represent the sum of these five categories.

**Table 2:** Diagnostic efficacy of different screening methods in the first trimester for CHD.

	Sensitivity (%)	Specificity (%)	Positive predictive value (%)	Negative predictive value (%)	AUC
4CV	34.09	99.96	93.75	98.77	0.670
4CV + 3VT	60.23	99.96	96.36	99.26	0.804
Four-section approach	67.05	99.96	96.58	99.33	0.835

A total of 176 fetuses were diagnosed with CHD. The accurate detection rates of fetal visceral heart position abnormality, inflow tract abnormality, outflow tract abnormality, aortic arch and its branches abnormality, and other heart abnormality were 100% (9/9), 73.9% (17/23), 62.3% (33/53), 73.6% (39/53), and 10.5% (4/38), respectively. CHD types with higher accurate diagnosis rates during early pregnancy include ectopic heart (2/2,100%), displaced heart (1/1, 100%), mirror-image dextrocardia (2/2, 100%), isomeric syndrome (4/4,100%), mitral atresia (1/1, 100%), hypoplastic left heart syndrome (3/3, 100%), tricuspid atresia (1/1, 100%), hypoplastic right heart syndrome (4/4, 100%), absence of semilunar valve (7/7, 100%), aortic atresia (1/1, 100%), aberrant right subclavian arteries (22/26, 84.6%), right aortic arch (7/8, 87.5%), transposition of the great artery (5/6, 83.3%), double outlet right ventricle (4/5, 80.0%), Ebstein's anomaly (2/3, 66.7%), tetralogy of Fallot (11/16, 68.8%), atrioventricular septal defect (6/9, 66.7%), pulmonary atresia (6/9, 66.7%), aortic arch stenosis (8/14, 57.1%), and double aortic arch (1/2, 50.0%). CHD that are more difficult to diagnose in early pregnancy include ventricular septal defect (4/30, 13.3%), pulmonary valve stenosis (0/10, 0%), pulmonary artery sling (0/2, 0%), anomalous pulmonary venous connection (0/2, 0%), retroaortic left innominate vein (0/1, 0%), myocardial insufficiency (0/1, 0%), coronary fistula (0/1, 0%), and rhabdomyoma (0/1, 0%). CHD in early pregnancy that indicate only minor cardiac abnormalities without a clear diagnosis include aortic arch dissection and main-pulmonary window.

### Missed diagnosis and misdiagnosis during early pregnancy

In this study, a total of 176 fetuses with CHD were identified by pathological and clinical verification, and 102 fetuses were accurately diagnosed during early pregnancy. Seventy-four fetuses were inconsistent with the verification results of fetal cardiac ultrasound during early pregnancy (including 22 fetuses with unreliable diagnosis caused by disease evolution and progression and 52 fetuses with inconsistent diagnosis due to missed misdiagnosis), as presented in Table 3.

**Table 3:** Inconsistency between first trimester ultrasonography and final diagnosis.

	Missed diagnosis and misdiagnosis lead to inconsistencies in diagnosis			The progression of the disease leads to inconsistent diagnosis		
	Serial number	Early pregnancy diagnosis	Final diagnosis	Serial number	Early pregnancy diagnosis	Final diagnosis
Abnormal inflow tract	1	Normal^a^	P-AVSD^a^	1	Normal	MS^b^
** **	2	Normal^c^	C-AVSD^c^	2	Normal	TVD^d^
** **	3	HLHS^e^	C-AVSD^e^			
** **	4	Normal^a^	Ebstein^a^			
Abnormal inflow tract	5	Normal^a^	TOF^a^	3–11	Normal	PS^b,f^
** **	6	Normal^a^	TOF^a^	12	VSD	TOF^f^
** **	7	PA < AO^e^	TOF^e^	13	VSD	TOF^f^
** **	8	DORV^e^	TGA^e^	14	VSD	DORV^f^
** **	9	PTA^e^	PA-IVS^e^	15	TR	PS^f^
** **				16	PA < AO	PA-IVS^f^
** **				17	TOF	PA-VSD^d^
Abnormal aortic arch and its branches	10–13	Normal^c^	ARSA^c^	18–20	Normal	CoA^b,f^
** **	14	Normal^c^	RAA^c^			
** **	15	TR^e^	CoA^e^			
** **	16/17	LV < RV, PLSVC^e^	CoA^e^			
** **	18	PA > AO^e^	IAA^e^			
** **	19	PA < AO^e^	APW^e^			
** **	20	RAA^c^	DAA^c^			
Others	21–45	Normal^a^	VSD^a^	21	Normal	CR^f^
	46/47	Normal^a^	TAPVC^a^	22	Normal	NVM^d^
	48	Normal^a^	RLINV^a^			
	49	Normal^a^	CAF^a^			
	50	Normal^a^	PAS^a^			
	51	L-LU > R-LU	PAS with UHPA^e^			
		Displacement of the heart				
	52	AVSD^e^	VSD^e^			
Normal	53–55	VSD^a^	Normal^a^			
	56	LV < RV^a^	Normal^a^			

AVSD, atrioventricular septal defects; C-AVSD, complete atrioventricular septal defect; TAPVC, total anomalous pulmonary venous connection; DORV, double outlet right ventricle; COA, coarctation of aorta; MS, mitral stenosis; VSD, ventricular septal defect; HLHS, hypoplastic left heart syndrome; RAA, right aortic arch; ARSA, aberrant right subclavian artery; TOF, tetralogy of Fallot; TGA, transposition of great arteries; P-AVSD, partial atrioventricular septal defect; PA-IVS, pulmonary atresia with intact ventricular septum; DAA, double aortic arch; IAA, interrupted aortic arch; APW, aortopulmonary window; PAS, pulmonary artery sling; RLINV, retroaortic left innominate vein; CAF, coronary artery fistula; RAA, right aortic arch; CAT, common arterial trunk; TR, tricuspid regurgitation; PA, pulmonary artery; AO, aorta; UHPA, unilateral hypoplasia of pulmonary arteries; L-LU, left lung; R-LU, right lung; PLSVC, persistent left superior vena cava; LV, left ventricle; RV, right ventricle; TVD, tricuspid valve dysplasia; CR, cardiac rhabdomyomas; NVM, non-compaction of ventricular myocardium.

^a^ The missed and misdiagnosis caused by the limitation of cardiac ultrasound in early pregnancy.

^b^ Progressed after birth.

^c^ Misdiagnosis due to equipment adjustment problems or poor fetal position.

^d^ Progressed from the second to the third trimester.

^e^ Misdiagnosis due to lack of knowledge.

^f^ Progressed from the first to the second trimester.

A total of 22 fetuses with inconsistent diagnosis caused by the progression of CHD in the uterus were reported, among which 16 fetuses (72.7%, 16/22) progressed from the first to the second trimester, three fetuses (13.6%, 3/22) progressed from the second to the third trimester, and three fetuses (13.6%, 3/22) progressed after birth. Among the 22 fetuses, 12 fetuses (54.6%) had valvular diseases, eight fetuses (36.4%) had arterial obstructive diseases, one fetus (4.5%) had cardiac tumors, and one fetus (4.5%) had cardiomyopathy.

Three types of missed misdiagnosis are noted: Class I is the limitation of cardiac ultrasound limited in the first trimester. Class II is impaired subjective cognition leading to missed misdiagnosis. Class III has missed misdiagnosis due to instrument adjustment problems or poor fetal position. A total of 14 CHD types were detected in the second trimester. Pulmonary stenosis, total anomalous pulmonary venous connection, pulmonary artery sling, retroaortic left innominate vein, coronary artery fistula, and cardiac rhabdomyoma were the CHD types not detected in the first trimester. Two CHD types were detected in the third trimester, such as tricuspid valve dysplasia and non-compaction of ventricular myocardium. Both were not detected by ultrasound in the first trimester. Four types of CHD were detected in the neonatal period, among which ultrasound did not detect mitral stenosis during pregnancy.

### Abnormal imaging pattern of four-section approach

**Figure 4:**
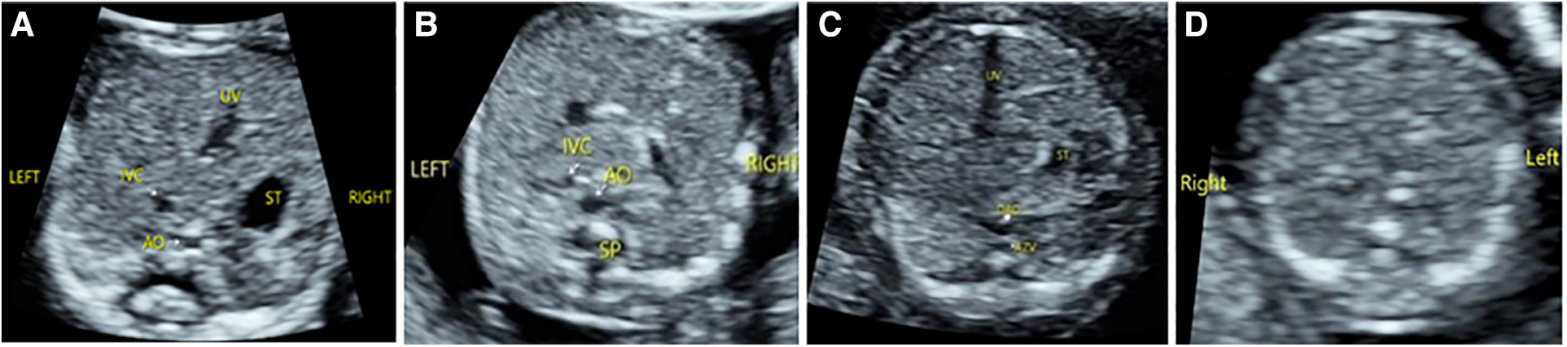
Abnormal image pattern of ultrasound in the upper abdominal transverse section. (**A**) Patterns of UAb-1: The stomach is on the right side of the abdomen, the superior vena cava is on the left front of the spine, and the abdominal aorta is on the right front of the spine. (**B**) Patterns of UAb-2: The stomach is small and located near the midline, while the superior vena cava is on the same side of the spine as the abdominal aorta. (**C**) Patterns of UAb-3: The stomach is small and located near the midline, and the dilated (hemiazygos vein) azygos vein is seen behind the right (left) of the descending aorta. (**D**) Patterns of UAb-4: The stomach is not seen on the transverse section of the upper abdomen.

**Figure 5:**
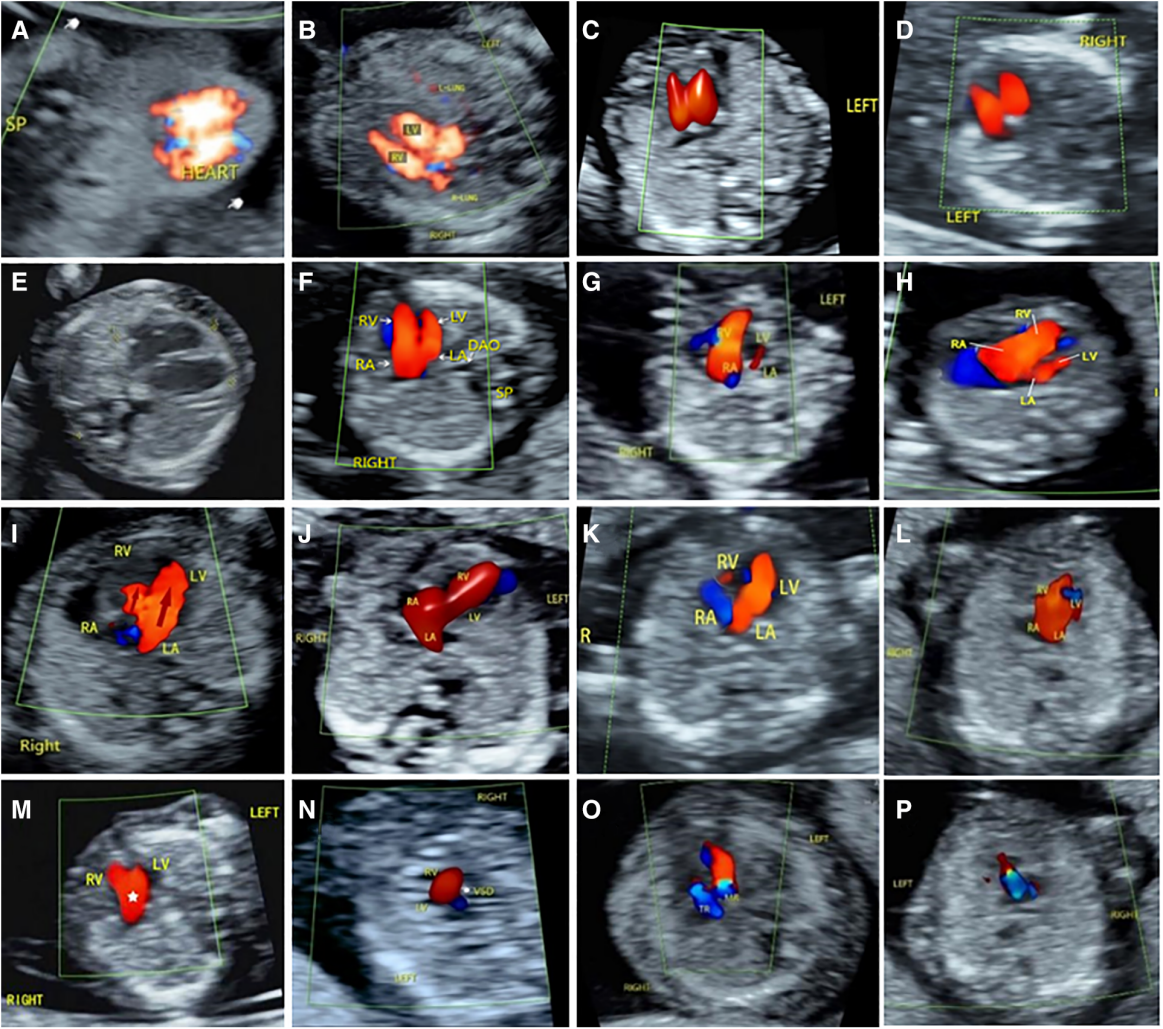
Abnormal ultrasound image pattern of 4CV section. (**A**) Patterns of 4CV-1 showed that the heart is partially or completely located outside the chest cavity. (**B**) Patterns of 4CV-2 showed displacement of heart. (**C**) Patterns of 4CV-3: the cardiac axis is abnormally shifted to the forward. (**D**) Patterns of 4CV-4: The cardiac axis is abnormally shifted to the right. (**E**) Patterns of 4CV-5 indicate an increase in cardiothoracic ratio. (**F**) Patterns of 4CV-6, filling of both ventricles, but the left ventricle appears smaller. (**G**) Patterns of 4CV-7: The left heart is spherically enlarged, and the filling of left ventricle appears smaller. (**H**) Patterns of 4CV-8: The filling of the right heart increased obviously. (**I**) Patterns of 4CV-9, filling of both ventricles, but the right ventricle appears very smaller. (**J**) Patterns of 4CV-10, filling of only the right ventricle. (**K**) Patterns of 4CV-11, filling of only the left ventricle. (**L**) Patterns of 4CV-12 showed a central singular inflow. (**M**) Patterns of 4CV-13 indicate “Y”-shaped filling of the ventricular inflow tract during diastole. (**N**) Patterns of 4CV-14 indicate a ventricular septal defect. (**O**) Patterns of 4CV-15, mitral or tricuspid regurgitation. (**P**) Patterns of 4CV-16, common valve regurgitation in atrioventricular septal defect.

**Figure 6:**
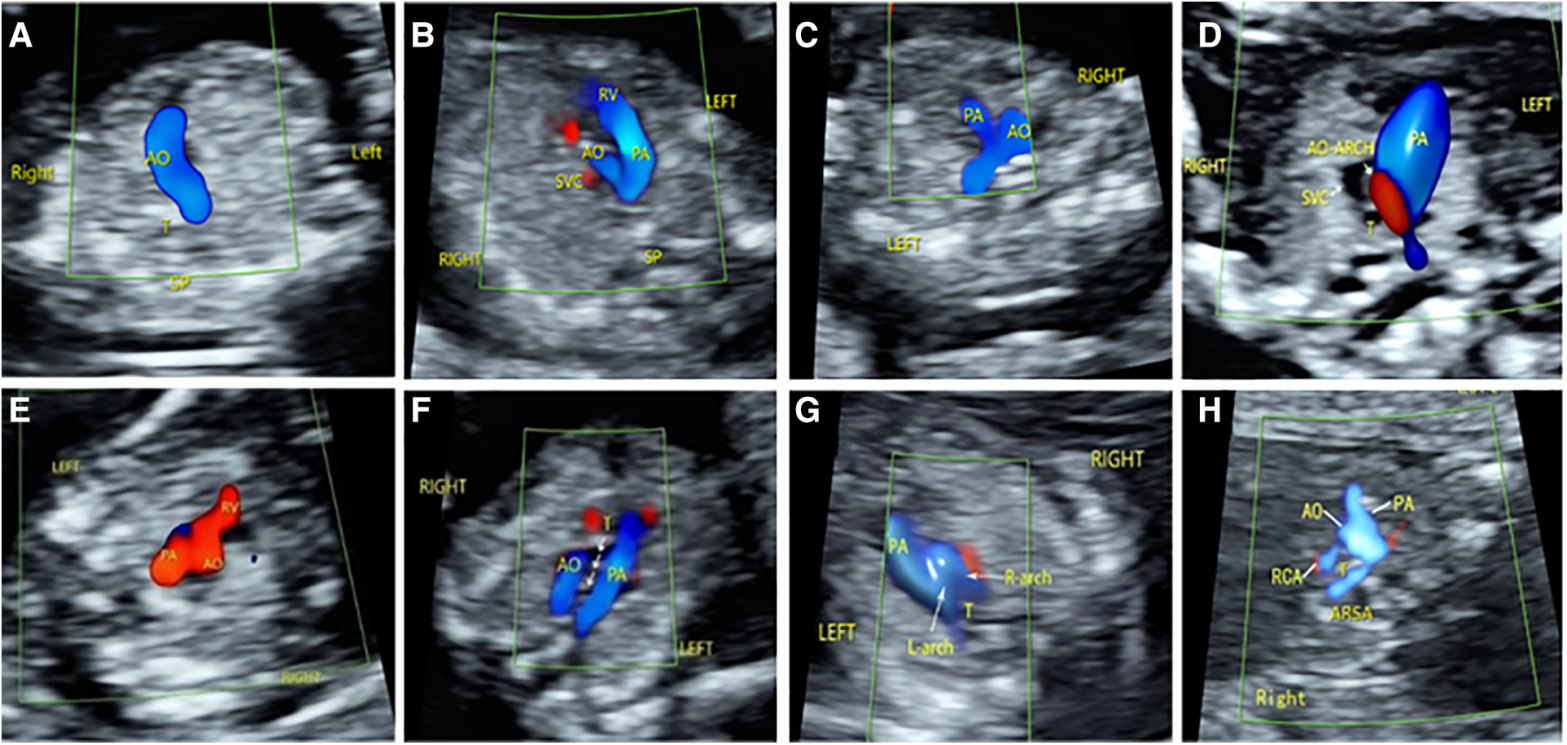
Abnormal ultrasound image pattern of 3VT section. (**A**) Patterns of 3VT-1, one vessel with a curved course or a straight course. (**B**) Patterns of 3VT-2: The aorta was smaller than the pulmonary artery. (**C**) Patterns of 3VT-3: The pulmonary artery was smaller than the aorta. (**D**) The aorta was smaller than the pulmonary artery, and reverse flow was observed in the aortic arch. (**E**) The “in–out sign” of the aorta and/or pulmonary artery. (**F**) Patterns of 3VT-6, two large arteries forming a “U” sign around the trachea. (**G**) Patterns of 3VT-7, two large arteries forming an “O” sign around the trachea. (**G**) Patterns of 3VT-8: The subclavian artery and aorta together form a “C” sign around the trachea. (**H**) Patterns of 3VT-8: The subclavian artery originates from the origin of the descending aorta and runs behind the trachea to form a “C”-type vascular ring.

**Figure 7:**
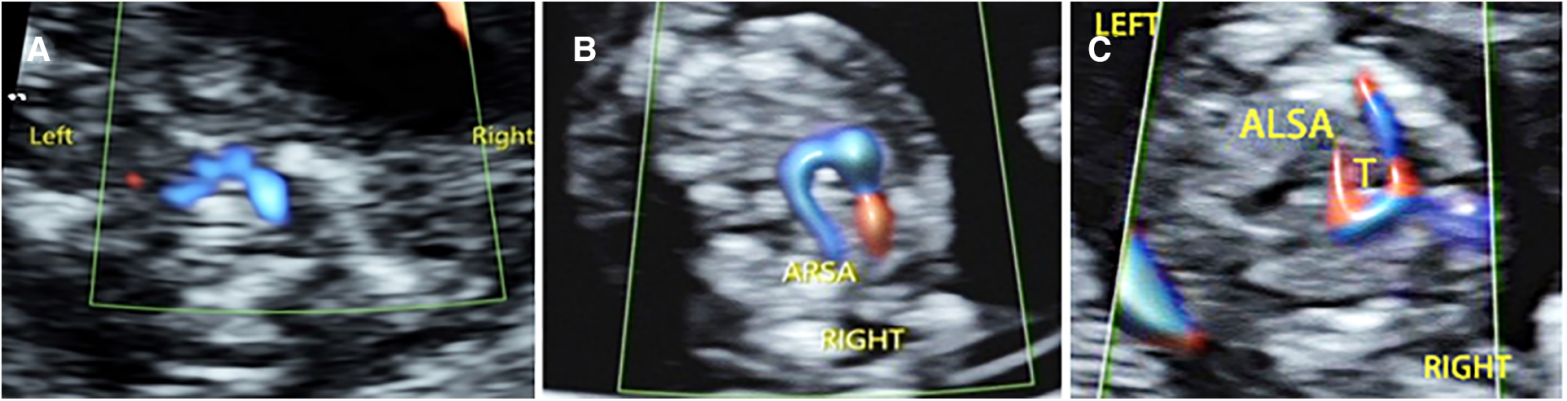
Abnormal ultrasound image pattern of the bilateral subclavian artery section. (**A**) Normal bilateral subclavian arteries are in front of the trachea, and they form a “bow” structure. (**B**) BSa-1 pattern: The right subclavian artery originates from the descending aorta, bypasses the trachea, and then runs to the right. (**C**) BSa-2 pattern shows that the left subclavian artery originates from the descending aorta and is circumvented behind the trachea to the left.

Among the 176 fetuses with CHD, 10 fetuses had an abnormal upper abdominal transverse section. According to the integrity of the fetal abdominal wall and the spatial position of four U-shaped structures (gastric bleb, inferior vena cava, aorta, and spine), four abnormal image patterns of UAb transverse section were found, which could be seen in normal cases, situs inversus, left isomerism syndrome, and right isomerism syndrome (Figure 4). According to the location, size, cardiac axis, the central crisscross structure of the heart and the size and direction of blood flow bundles, 16 kinds of 4CV section abnormal patterns were found (Figure 5). According to the number of large arteries, the relationship between the pulmonary artery and the aorta, the diameter of the pulmonary artery and the aorta, and the direction of blood flow between the ductus arteriosus and the aortic arch, eight abnormal patterns of 3VT section were found (Figure 6). According to the course of bilateral subclavian arteries, two abnormal patterns of BSa sections were found (Figure 7).

## Discussion

Based on the 4CV section and 3VT section, the upper abdominal transverse section and bilateral subclavian artery section were added. The upper abdominal transverse section is the first section of the heart segment analysis and should be considered as part of the cardiac screening during early pregnancy. The bilateral subclavian artery section complements the 3VT section, providing additional anatomical information about the aortic arch and its branches. Four-section approach has a high detection rate for fetal visceral heart position abnormalities, inflow tract abnormalities, outflow tract abnormalities, and aortic arch and branch abnormalities. In our study, 67.05% of all CHD were diagnosed in early pregnancy, considering that ventricular septal defect was not included in most studies. The sensitivity and specificity reached 77.55% and 99.98% after excluding the ventricular septal defect, and this is higher than other studies (15). It is proved that the four-section approach adopted by our center in early pregnancy is reliable and effective for diagnosing CHD.

With the improvement of ultrasonic instrument resolution and blood flow imaging mode, the imaging ability of cardiac structure in early pregnancy has been significantly optimized. Previous studies have described the most common abnormal blood flow patterns of the 4CV section and 3VT section in fetuses of 11–13^+6^ weeks by two-section method (14), which will significantly promote the development of cardiac screening in early pregnancy. This study analyzed CHD’s ultrasonic characterizations based on the four-section approach at 11–13^+6^ weeks and further complemented the pattern of abnormal images. We summarized the four abnormal image patterns in the upper abdominal transverse section, representing the four types of abnormalities. The observation of the size and location of the gastric vesicle in the transverse section of the upper abdomen and the location of the inferior vena cava and abdominal aorta plays an important role in determining the position of the viscera, which can assist in the diagnosis of the isomeric syndrome (20, 21). 4CV section abnormalities were most common in the inflow tract of the heart. The abnormal image patterns of 4CV section can be divided into eight categories. Irregular heart size is mainly manifested as the increased cardiothoracic ratio, and heart failure caused by severe valvular regurgitation is expected in early pregnancy. The abnormal balance of left and right ventricular blood flow bundles can be divided into right heart dominant and left heart dominant. Right heart dominance is mostly caused by coarctation of the aortic arch, hypoplastic aortic arch, or interrupted aortic arch. In addition, tricuspid valve developmental abnormalities such as Ebstein’s anomaly may contribute to the right heart-dominant flow pattern in early pregnancy. Some studies have shown that right heart dominance is also seen in atrioventricular junction inconsistencies or related to partial proper ventricular double outlet (22). Left heart dominance is common in right ventricular dysplasia, but it is also a sign of severe aortic stenosis (23). Since some valvular pathologies are progressive, it is essential to regularly review and monitor the asymmetry of the left and right ventricular flow bundle widths. A single blood flow bundle in the heart is a manifestation of severe CHD (14). The single arterial blood flow bundle is vertically oriented, suggesting a pulmonary bundle, which is common in HLHS; if the single arterial blood flow bundle is curved to the side, it suggests an aortic arch blood flow bundle, which is common in conotruncal defects. Due to the aliasing of left and right ventricular blood flow bundles, it is difficult to show the transseptal blood flow bundles of the ventricular septal defect in early pregnancy. If suspicious cases are found based on grayscale ultrasound, multi-section scanning and follow-up are needed. Mitral and/or tricuspid valve regurgitation are often transient, and mild atrioventricular valve regurgitation can be easily missed by color Doppler observation alone. Therefore, it is important to routinely use pulsed Doppler for standard sampling. When 4CV section abnormalities are found, further careful evaluation of 3VT section should be performed.

In this study, 3VT section abnormalities were most seen in outflow tract malformations. The abnormal image patterns can be divided into eight categories. The single arterial blood flow bundle can be divided into two types according to its course (24). The single arterial blood flow bundle is vertically oriented, suggesting a pulmonary bundle, which is common in HLHS; if the single arterial blood flow bundle is curved to the side, it suggests an aortic arch blood flow bundle, which is common in conotruncal defects. When a single blood flow bundle is found, the diagnosis should be further refined by observing the blood flow of the ductus arteriosus and the connection between the large artery and the ventricle. The proportion of blood flow bundles can also be divided into two types. A small aortic blood flow bundle, especially in 4CV views with right heart–dominant flow, suggests a narrowing of the aortic arch. Conversely, a narrow pulmonary artery blood flow bundle that forms a “Y” blood flow bundle with a widened aorta may indicate pulmonary stenosis, and it is most common in tetralogy of Fallot and can also be seen in type IV double outlet right ventricle and simple pulmonary artery stenosis (11). In atypical cases, ventricular septal defect and MPA/AO ratio can be suggested, and the diagnosis can be improved in the follow-up review (25). “Back and forth” flow bundles are characteristic of the absence of a semilunar valve, which can be seen in the absence of aortic valve, pulmonary valve, or bisemilunar valve. Some scholars call it “in and out” (26, 27). “U”-, “0”-, and “C”-shaped blood flow bundles are found in vascular rings formed by the abnormal aortic arch and its branches. It is difficult to accurately identify them in early pregnancy. To summarize, after obtaining a 3VT section under optimal conditions, if a “V” sign is not observed on this view, which is not formed by the ductus and aortic arch, careful analysis of the abnormal pattern is needed, and multiple sections should further confirm the diagnosis. The bilateral subclavian artery section is an effective complement to 3VT section. Because the angle between the acoustic beam and the flow direction of the right subclavian artery is too large, it is difficult to show the aberrant subclavian artery on the 3VT section. In contrast, the transverse “arch,” like the structure of the bilateral subclavian artery, is easy to identify. By observing the spatial location of bilateral subclavian arteries and their relationship with the trachea, their abnormal image patterns can be divided into two categories. Failure to show this typical structure suggests the presence of an aberrant subclavian artery.

Recognizing abnormal image patterns in different sections benefits the rapid recognition and accurate diagnosis of fetal CHD in early pregnancy. However, it should be noted that different abnormal image patterns may appear in the same disease, and the same image patterns may also appear in various conditions. When abnormalities are found, the types of CHD should be determined by combining abnormal image patterns on different sections and using multi-section scanning. In addition, the ultrasonic beam incident angle, Doppler gain, pulse repetition rate, and wall filtering are the key to obtaining the standard image, which should be dynamically adjusted according to the observation object and purpose.

It should be noted that, under the influence of multiple factors such as the examiner's operating experience, instrument resolution, pregnant women's BMI, and gestational age (28–31), missed diagnosis and misdiagnosis are still the main reasons for the inconsistency between early fetal echocardiography and subsequent verification. By classifying and summarizing different causes of missed diagnosis and misdiagnosis, cardiac screening in early pregnancy and prenatal consultation will be beneficial. In this study, the cases of the 56 fetuses with CHD were found to be missed diagnoses and misdiagnoses by ultrasound during early pregnancy. The causes of missed diagnosis and misdiagnosis can be divided into three categories: Category I was the fetal heart during early pregnancy and the limitations of the ultrasound equipment, Category II was the lack of subjective understanding, and Category III was caused by instrument adjustment problems or poor fetal position. Class I included ventricular septal defect, mild tetralogy of Fallot, and anomalous pulmonary venous connection. The early diagnosis of some CHD is still a significant challenge due to objective reasons such as small fetal hearts in early pregnancy and the resolution of ultrasonic instruments. The false-positive and false-negative rates of VSD in early pregnancy are both high because the ventricular septum is prone to produce echo loss artifacts on the 4CV section and the color Doppler left and right ventricular flow tracts are inclined to produce aliasing artifacts (32, 33). According to the study by Hutchinson (34), only 5% of pulmonary veins in 12–13 weeks of fetuses can be observed by conventional ultrasound mode. With the high-resolution blood flow imaging technology (31), the detection of pulmonary veins in early pregnancy is expected to be realized. A total of 11 fetuses in class II were misdiagnosed due to a lack of understanding. For example, a small left heart is one of the indications of coarctation of the aortic arch, but it can also be seen in normal fetuses or aneuploidies. Therefore, based on the color Doppler ultrasound, when fetal heart abnormalities or minor abnormalities are found, the cardiac structure should be carefully evaluated by two-dimensional ultrasound and carefully judge the two large arteries and the relationship between the large arteries and the ventricle. Transvaginal ultrasound is an essential adjunct for detailed anatomical observation (34). In Class III, seven fetuses were misdiagnosed due to instrument adjustment or poor fetal position. For example, excessive color Doppler gain in 4CV section leads to the missed diagnosis of atrioventricular septal defect. Therefore, to avoid missed diagnosis and misdiagnosis of Class III, we should pay attention to the adjustment of Doppler gain and ultrasonic incidence angle during the fetal heart examination in early pregnancy.

The intrauterine progression of the fetal heart is another major reason for inconsistency between ultrasound examination in the first trimester and subsequent verification. With the advancement of technology, we are able to diagnose various forms of CHD at different stages before birth, which provides a prerequisite for observing the hemodynamic changes and intrauterine progression of fetal CHD from early to late pregnancy. In this study, we analyzed intrauterine progression of the fetal heart at different stages and found that progression between 11 and 24 weeks was the most common. The reasons may be considered as follows. On the one hand, fetal weight and heart will experience the maximum growth throughout the entire pregnancy period. Therefore, a decrease in blood flow to the ventricles or major arteries may seriously affect the development and growth of the corresponding ventricular structures (22, 35), while on the other hand, most fetuses with congenital heart disease are induced after early diagnosis, making it unable to observe the complete natural progression. This study recorded the progression of CHD in 22 fetuses in different periods, including inflow tract obstructive diseases, outflow tract obstructive diseases, aortic arch obstruction, cardiomyopathy, and heart tumors, among which heart valve diseases are the most common, followed by arterial obstructive diseases. The intrauterine progression of CHD increases the uncertainty of the disease and poses a huge challenge to clinical consultation. Therefore, it is crucial to understand the occurrence and potential development trends of fetal heart malformations for prognosis consultation, intrauterine treatment, and perinatal management. Once the fetal heart malformation is diagnosed in prenatal testing, the possibility of the progression must be considered.

Due to factors such as ultrasound technology level, fetal position, fetal circulation state, and maternal sound window, the missed diagnosis and misdiagnosis of fetal CHD in early pregnancy may be caused (17). In addition, intrauterine progression may occur in some CHD cases, and the structural and hemodynamic changes of the fetal heart can also be significantly different before and after delivery. Due to these particularities, there are uncertain factors in the prenatal examination of fetal heart malformations (33, 36). Therefore, a routine prenatal ultrasound examination cannot rule out the possibility of subsequent CHD. Attention should be paid to sequential ultrasound examination and follow-up management of fetal echocardiography to maximize the early diagnosis rate of CHD.

## Limitation

This study has some limitations. First, although we screen all newborns for congenital heart disease after birth, not all newborns receive echocardiography. Second, only fetuses with the final verification results in our center are included, while fetuses that have been diagnosed with CHD but have yet to be further confirmed in our center are excluded.

## Conclusion

The incidence of fetal CHD was 1.85%, and about 67.05% of CHD could be detected by a four-section approach during early pregnancy. The four-section approach has a high detection rate for the abnormal position of the fetal visceral heart, abnormal inflow tract, abnormal outflow tract, and abnormal aortic arch and branches. Different types of CHD in the first-trimester fetuses have characteristic manifestations on the four-section scanning method, and the abnormal image patterns of CHD ultrasound in each section are summarized and familiarized, which is conducive to early pregnancy CHD rapid identification and diagnosis.

## Data Availability

The original contributions presented in the study are included in the article, further inquiries can be directed to the corresponding authors.
